# Fusobacterium nucleatum: More Than Just an Oral Anaerobe

**DOI:** 10.7759/cureus.26989

**Published:** 2022-07-18

**Authors:** Akash Patel, Monica Patel, Joseph Glowacki

**Affiliations:** 1 Internal Medicine, St. Mary Medical Center, Langhorne, USA; 2 Internal Medicine, Temple University Hospital, Philadelphia, USA; 3 Internal Medicine, Rowan University School of Medicine, Stratford, USA

**Keywords:** anaerobes of clinical importance, colon cancer surveillance, periodontal disease, brain abscess, fusobacterium nucelatum

## Abstract

*Fusobacterium nucleatum* is a strict anaerobe that is indigenous to the human oral cavity, where it coexists with more than 500 other species. It is associated with paranasal sinus, odontogenic, and pulmonary infections. In literature, cases of *Fusobacterium nucleatum* are rare. Here we report a case of a patient with multiple brain abscesses caused by *Fusobacterium nucleatum*. This case report looks to assist clinicians in determining the true etiology of this organism, which can change patient management based on the current literature review and similar case studies.

## Introduction

In western countries, developing a brain abscess is rare with its incidence at 1% to 2%. Cultures typically show polymicrobial infection in 30% to 60% of cases with streptococci being the most common organism. In about 40% of cases, anaerobic bacteria are responsible for the development of an abscess. Of these anaerobes, those belonging to the genera *Fusobacterium* and *Peptostreptococcus* are often the culprits [1]. Of the *Fusobacterium* genus, *F. nucleatum* and *F. necrophorum* are the most common species seen clinically. *Fusobacterium nucleatum* is a strict anaerobe that is indigenous to the human oral cavity, where it coexists with more than 500 other species [2]. It is associated with paranasal sinus, odontogenic, and pulmonary infections, but is not commonly seen causing central nervous system (CNS) infections. Here, we report a case of a patient with multiple brain abscesses caused by *F. nucleatum*. 

## Case presentation

**Figure 1:**
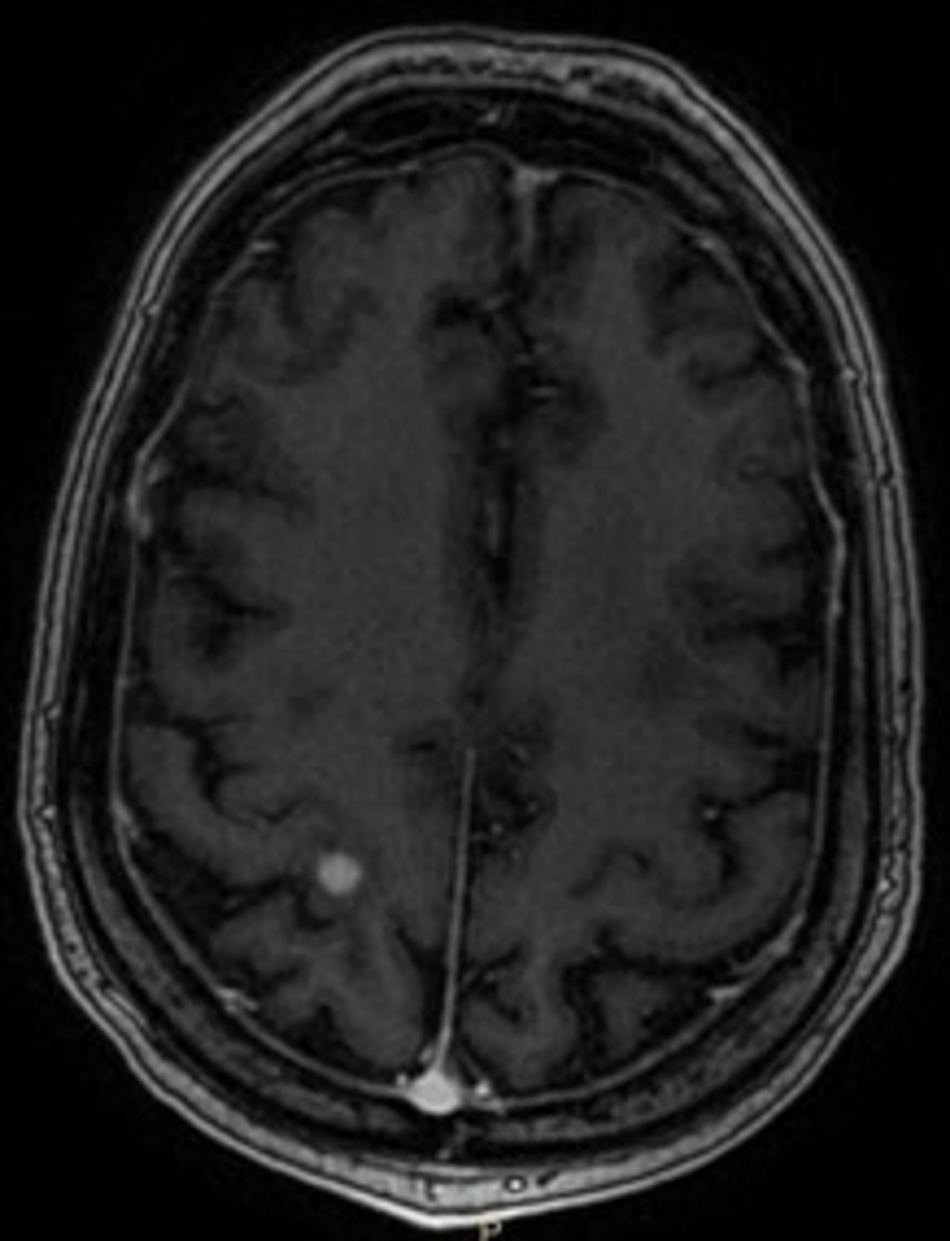
MRI brain with contrast, T1-weighted image showing 0.7 cm high right parietal lobe lesion

**Figure 2:**
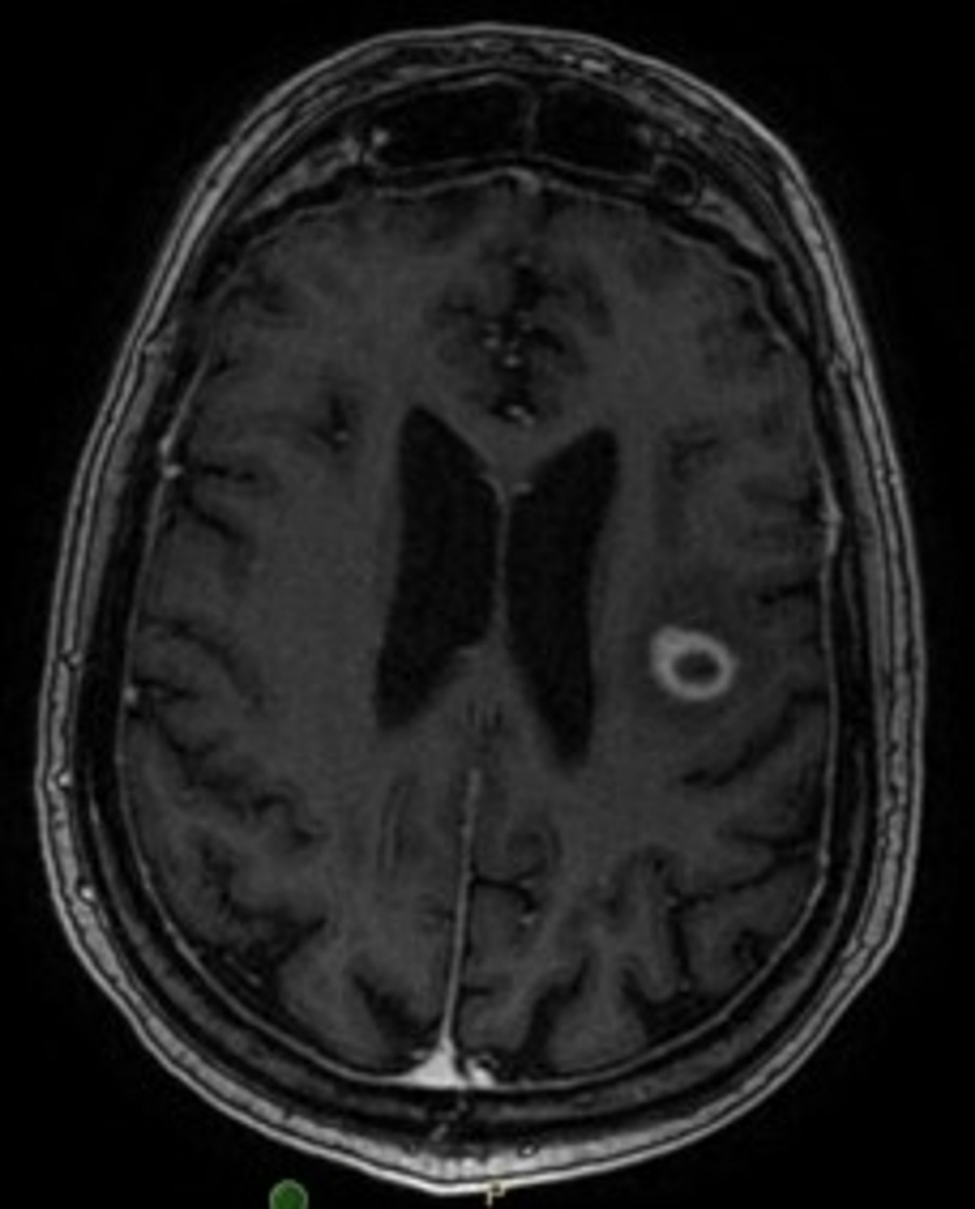
MRI brain with contrast, T1-weighted image showing 1.5 cm left frontal lobe lesion

**Figure 3:**
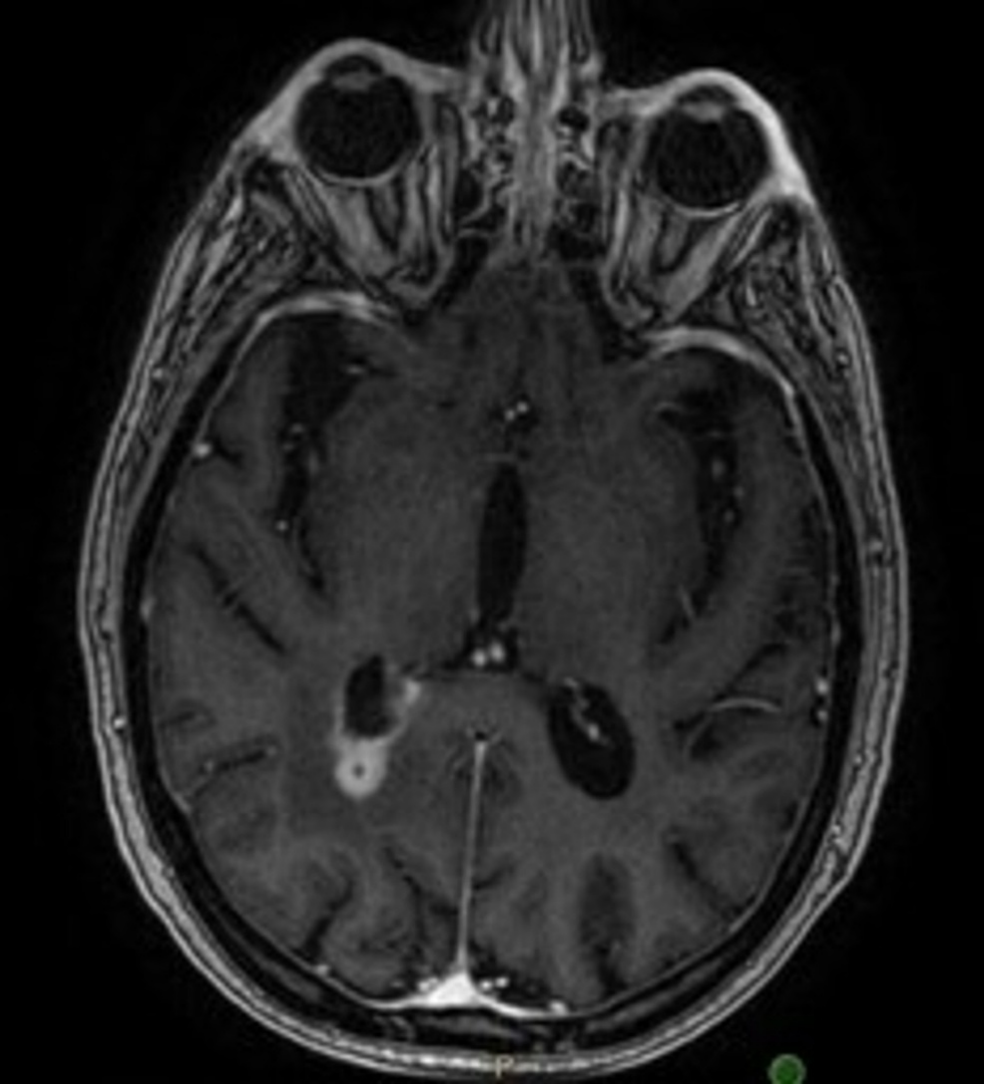
MRI brain with contrast, T1-weighted image showing 1.1 cm right parieto-occipital adjacent to the atrium of the right lateral ventricle

**Figure 4:**
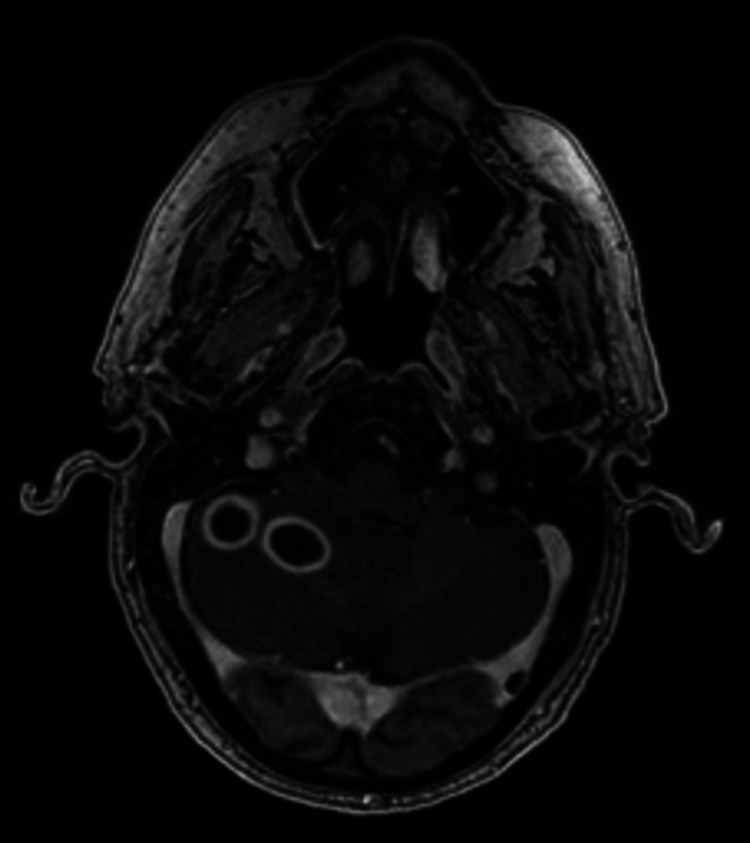
MRI brain with contrast, T1-weighted image showing 2.0 cm and 1.7 cm lesions within the right cerebellar hemisphere

A 63-year-old male with unsteady gait and mechanical falls for three weeks in duration presented to us. On review of systems, the patient admitted to frequent headaches and upper respiratory symptoms including rhinorrhea and cough over two weeks. The patient has a history of alcohol and tobacco use. On exam, the patient appeared disheveled, had poor oral dentition with missing teeth and multiple dental caries, and had a positive pronator drift test. Comprehensive metabolic panel (CMP), complete blood count (CBC), erythrocyte sedimentation rate (ESR), and c-reactive protein (CRP) were nonsignificant and urodynamic studies (UDS) were negative. A CT of the head and computed tomography angiography (CTA) of the head and neck were negative. Due to the chronic respiratory complaints, a CT chest was performed and revealed a 9.0 x 2.5 cm pleural mass in the right lower lobe. An MRI of the brain showed multiple peripherally enhancing lesions throughout (Figures 1-4). Initially, it was suspected the patient had primary lung carcinoma with metastasis to the brain given the imaging findings. The patient underwent a CT-guided right lower lobe pleural mass biopsy which showed scattered mesothelial cells and granulation tissue favoring a scar. Due to biopsy results indicating an inflammatory process and not a neoplastic process, the patient then underwent right hemicraniectomy where multiple abscesses were found and drained. Cultures grew *F. nucleatum* which we suspect was secondary to hematological spread from an oral infection and the patient completed six weeks of antibiotics with ceftriaxone 2 g every 12 hours (q12H) and metronidazole 500 mg every 8 hours (q8H). The patient’s gait difficulties were resolved and he was discharged home with a follow-up with gastroenterology and without the need for any physical therapy.

## Discussion

*Fusobacterium nucleatum* is mainly associated with orthodontic infections but is also less commonly seen in other infections such as “sinusitis, pulmonary infections, intra-abdominal infections, i.e. appendicitis and liver abscesses, and it was [also] detected in a psoas abscess, septic arthritis, osteomyelitis and endocarditis” [2]. Recent studies have also shown that* F. nucleatum* may also play a role in colorectal cancers. Despite the role of *F. nucleatum* in multiple different disease processes, there have been cases where the source of* F. nucleatum* remains a mystery and is presumed to be due to a periodontal source. *Fusobacterium nucleatum* is considered a mutualist, infectious agent, and oncogenic microorganism [3]. It has been shown to play an integral role in the development of biofilms. Due to its elongated shape, it has the unique ability to function as a bridging organism that can bind to other oral bacteria via different surface adhesins to help create and maintain a biofilm. This role with other organisms allows it to propagate periodontal disease in humans. *Fusobacterium nucleatum* can increase the invasive potential of other microorganisms such as *Porphyromonas gingivalis* to avoid destruction by the adaptive immune system and by creating a pro-inflammatory environment that eventually leads to worsening dental disease.

Recently, *F. nucleatum* has also been implicated in its role in colorectal cancer. This should come as no surprise since we have seen the association between microbes and carcinoma in the past. For example, human papillomavirus infection propagates mutational changes that lead to cervical cancer. Similarly, *Streptococcus bovis* bacteremia is a harbinger of colorectal cancer, which is now what new research is revealing about *F. nucleatum* [3]. Two studies by Kostic et al. and Castellarin et al. have compared the amount of* F. nucleatum* genetic material in healthy tissue versus tumor tissue showing that *Fusobacterium* genetic material was significantly higher in tumor tissue; this was most significant in colorectal tumor tissue [4]. Determining the true mechanisms behind these observations still remains ongoing.

Despite the current surge in research regarding* F. nucleatum* and the critical role it plays in a multitude of pathologies, no unified workup exists currently for patients found to have *F. nucleatum* bacteremia without an obvious source. Prior to labeling the patient with “poor oral hygiene,” which is commonly done, new research on* F. nucleatum* indicates that a more extensive workup is warranted. Multiple studies have noted an association between *F. nucleatum* and colorectal cancer [5]; however, there is not a defined recommendation for colon cancer screening when* F. nucleatum* has been identified. On the other hand, when *Streptococcus gallolyticus*, a pathogen that is also associated with colorectal cancer, is the source of bacteremia and/or infective endocarditis, a prompt follow-up with colonoscopy is strongly recommended [6-7]. Similarly, in patients with blood cultures that are positive for *F. nucleatum*, we recommend close follow-up with a gastroenterologist to evaluate the patient’s personal risk for colorectal cancer and to consider colonoscopy and testing for tumor markers carcinoembryonic antigen (CEA) and cancer antigen 19-9 (CA 19-9) if warranted. 

In addition, *Fusobacterium* has the potential to cause metastatic infections due to its embolic characteristics. Bacteremia or infection at a primary site can lead to head, neck, pulmonary, cardiac, and intra-abdominal involvement [8-9]. Because of this potential, we recommend that if persistent *Fusobacterium *bacteremia is present, further imaging with CT head/chest/abdomen/pelvis be considered to rule out a secondary localized source of infection. An MRI should also be considered for enhanced visualization of a potential abscess when there is high clinical suspicion despite a negative CT scan (as seen in this case) or if the patient is not improving or responding to the current treatment regimen. 

Although uncommon, *F. nucleatum* can also present as endocarditis [10]. Anaerobic bacteria account for 2% to 16% of all infective endocarditis cases with the head and neck being the most common origin for *Fusobacterium *[8]. An echocardiogram is warranted in patients when a source of infection is unable to be determined, when blood cultures are persistently positive, or when there is a new-onset heart murmur.

Treatment for brain abscesses due to *F. nucleatum* remains early surgical intervention. However, some cases were successfully treated conservatively with antibiotics alone [11]. The non-surgical approach may be preferred when there are multiple abscesses, if the abscesses are small in size, if the abscesses are located in an unfavorable position, or if there appears to be cerebritis. Yet, in most cases, once a source of infection is identified, surgical evaluation should be considered to assist with source control by drainage if there is concern that antibiotic therapy alone may be inadequate. 

If the results of the proposed workup above are negative, we believe then it is then safe to assume a dental source of infection. Nevertheless, due to the strong association of *Fusobacterium* with dental infections, we recommend all patients found to have *Fusobacterium* infections follow up with their dentists for a complete evaluation. 

## Conclusions

*Fusobacterium nucleatum* has become more frequently recognized as a pathogenic organism due to its embolic potential and propensity for abscess formation. It is most commonly associated with poor dentition, and odontogenic infections, but can affect multiple organ systems. It is also crucial to obtain the necessary studies in order to effectively identify possible metastatic infections; this includes pertinent labs, cultures, imaging to investigate for potential abscesses, and consideration for an echocardiogram. It is also imperative to have close follow-ups with medical specialists, including infectious diseases, and gastroenterology for colonoscopy given the close association between *F. nucleatum* and colorectal cancer, and with dentistry due to the presence of *Fusobacterium* within the human oral microflora. 
